# Supplementary figures and images for: Is grid therapy useful for all tumors and every grid block design?
Source: J Appl Clin Med Phys. 2016 Mar 8;17(2):206–19. doi: 10.1120/jacmp.v17i2.6015 (PMC5874944; doi:10.1120/jacmp.v17i2.6015)

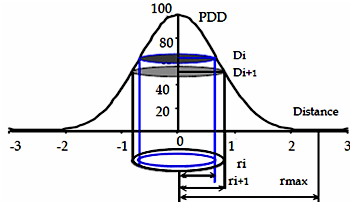

Supplement: Supplementary file 1 — Supplementary Material Files [file ACM2-17-206-s001.jpg]

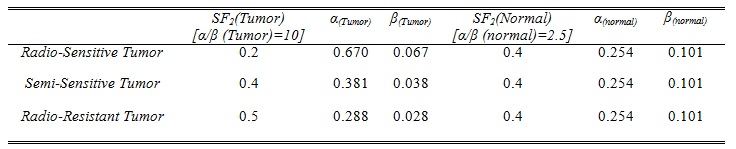

Supplement: Supplementary file 2 — Supplementary Material Files [file ACM2-17-206-s002.jpg]

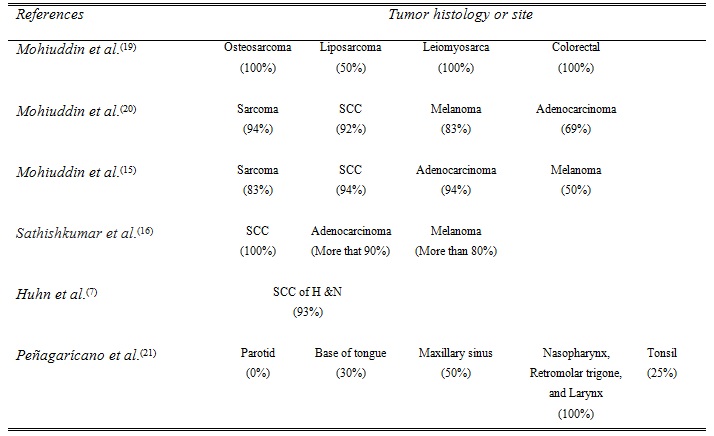

Supplement: Supplementary file 3 — Supplementary Material Files [file ACM2-17-206-s003.jpg]

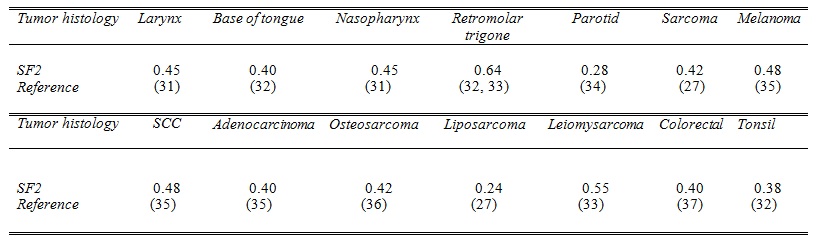

Supplement: Supplementary file 4 — Supplementary Material Files [file ACM2-17-206-s004.jpg]

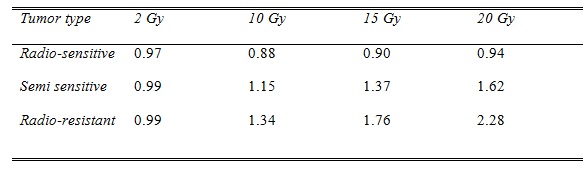

Supplement: Supplementary file 5 — Supplementary Material Files [file ACM2-17-206-s005.jpg]

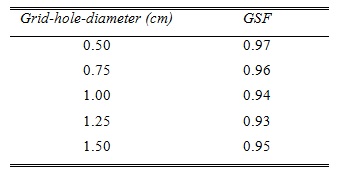

Supplement: Supplementary file 6 — Supplementary Material Files [file ACM2-17-206-s006.jpg]

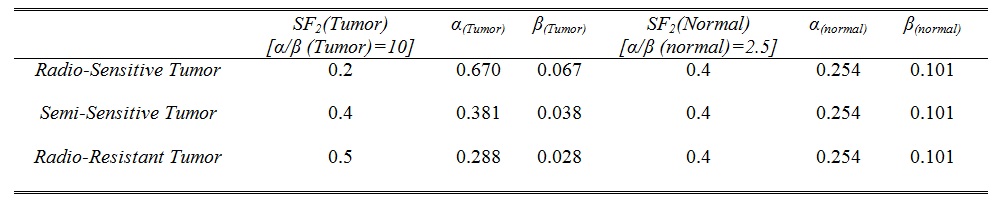

Supplement: Supplementary file 9 — Supplementary Material Files [file ACM2-17-206-s009.jpg]

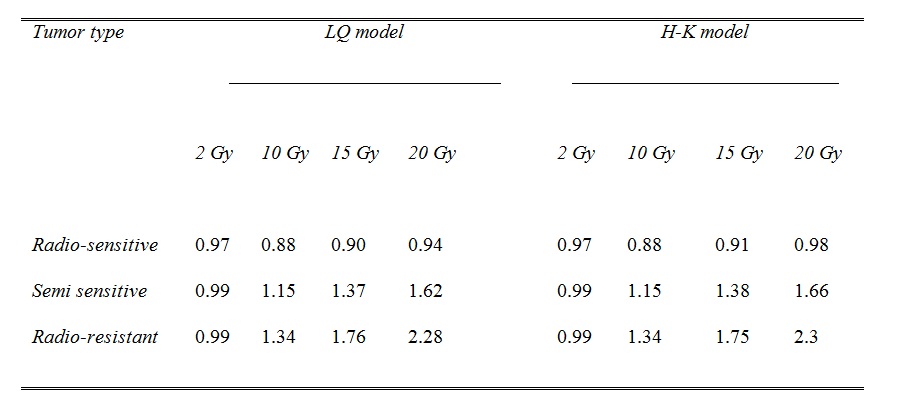

Supplement: Supplementary file 12 — Supplementary Material Files [file ACM2-17-206-s012.jpg]
