# Supplementary material for: Is grid therapy useful for all tumors and every grid block design?
Source: J Appl Clin Med Phys. 2016 Mar 8;17(2):206–19. doi: 10.1120/jacmp.v17i2.6015 (PMC5874944; doi:10.1120/jacmp.v17i2.6015)
Supplement: Supplementary file 7 — Supplementary Material Files [file ACM2-17-206-s007.docx]

**Is Grid Therapy Useful for all Tumors and Every Grid Block Design?**

**ABSTRACT**

Grid therapy is a treatment technique that has been introduced for patients with advanced bulky tumors. The purpose of this study is to investigate the effect of the radiation sensitivity of the tumors and the design of the Grid blocks on the clinical response of Grid therapy.

The Monte Carlo simulation technique is used to determine the dose distribution through a Grid block that was used for a Varian2100C linear accelerator. From the simulated dose profiles, the therapeutic ratio (TR) and the equivalent uniform dose (EUD) for different types of tumors with respect to their radiation sensitivities were calculated. These calculations were performed using the linear quadratic (LQ) and the Hug–Kellerer (H-K) models. The results of these calculations have been validated by comparison with the clinical responses of 232 patients from different publications, which were treated with Grid therapy. These published results for different tumor types were used to examine the correlation between tumor radio-sensitivity and the clinical response of Grid therapy. Moreover, the influence of Grid design on their clinical responses was investigated by using Monte Carlo simulations of Grid blocks with different hole-diameters and different center-to-center spacing.

The results of the theoretical models and clinical data indicated higher clinical responses for the Grid therapy on the patients with more radio-resistant tumors. The differences between TR values for radio-resistant cells and radio-sensitive cells at 20 Gy and 10 Gy doses were up to 50% and 30%, respectively. Interestingly, the differences between the TR values with LQ model and H-K model were less than 4%.

Moreover, the results from the Monte Carlo studies showed that Grid blocks with a hole-diameters of 1.0 cm and 1.25 cm may lead to about 19% higher TR relative to the Grids with hole-diameters smaller than 1.0 cm or larger than 1.25 cm ( with 95% confidence interval).

In summary, the results of this study indicate that Grid therapy is more effective for tumors with radio-resistant characteristics than radio-sensitive tumors.

Key words: Grid therapy, Clinical response, Geant4 simulation, Grid block design

PACS number: 87.55.-x

1. **INTRODUCTION**

Local control of bulky tumors with standard radiation therapy is a challenging topic because this treatment will involve a large volume of normal tissues ^(^[^1^](#_ENREF_1)^)^. Spatially fractionated radiation therapy (also known as Grid therapy), is a technique that has been introduced for treatment of patients with advanced bulky tumors ^(^[^2^](#_ENREF_2)^)^. In this technique, an open x-ray field is being converted to a set of pencil beam type radiation fields using an external block ^(^[^3^](#_ENREF_3)^)^. This block is normally made of lead or Cerrobend and also it could be created using an multi-leaf collimator (MLC) system in the linear accelerators ^(^[^3^](#_ENREF_3)^)^. Several investigators have reported that this technique has the advantage of a higher potential to repair normal tissues ^(^[^4-6^](#_ENREF_4)^)^.Different kinds of tumors had been treated using this technique and significant tumor responses have been observed without serious toxicities ^(^[^7^](#_ENREF_7)^)^.

Looking at the clinical experiences on the treatment of several hundred patients indicates dramatic effects of this radiotherapy technique on the regression of bulky tumors. Some radiotherapy centers have begun the routine use of spatially fractionated radiotherapy in the management of malignant disease for tumors larger than 6 cm. Despite these promising clinical outcomes, there is a lack of a protocol in the clinical references on selection of appropriate patient or disease for this type of treatment technique. Introduction of a guideline/recommendations for selecting the proper tumor type for this treatment technique would be highly beneficial. Furthermore, there is a missing recommendation for the geometrical design of a Grid block with appropriate hole-size (i.e. diameter) and hole center-to-center distances, which could provide an optimum therapeutic result.

In this project, the effectiveness of the Grid therapy is being evaluated for tumors with different radiation sensitivities. In addition, the impact of the geometrical design of the Grid blocks on their therapeutic ratios (TR) has been investigated. These investigations are based on the Monte Carlo simulations of the dose distribution of the Grid fields. The TR values are calculated using linear quadratic (LQ) model as well as the Hug–Kellerer (H-K) model. The integrity of these evaluations have been validated by comparison of the model based data with the clinically published values.

1. **MATERIALS AND METHODS**

Figure 1 shows the flow chart of the process of this project that would be described in details in the following subsections.

1. **Monte Carlo simulation**

In this study the Geant4 (Version 9.6.p02) Monte Carlo code^(^[^8^](#_ENREF_8)^)^ was used to simulate the photon spectrum of a 6 MV x-ray beam emitted by a Varian2100C linear accelerator. The simulations were performed in two steps: First, the accelerator head and the primary collimator were simulated to create the photon spectrum in a phase space (Phs) defined before the Jaws (Figure 2A). A total number of 10^9^ events were generated from the initial electron source to collect 50 million particles in the phase space as a scored plane with dimension of 40 ×40 ×0.2 cm^3^. The second part of the simulations included the Phs file that served as a source for simulating the dose distribution in the water phantom located after the Grid block. A 1 mm range cut-off in water was selected which corresponds to a 350 keV energy cut-off for electrons and positrons, and a 5 keV energy cut-off for photons. The water phantom that was used for these simulations had a dimension of 30×30×30 cm^3^. The center of the water phantom was modeled to be along the central axis of the beam. This phantom was divided into a set of voxels with dimensions of 2 × 2 × 2 mm^3^. The accuracy of the simulation was verified by comparison of the simulated data for a 10×10 cm^2^ open field size with the experimental data obtained with the same field geometry. The measurements were performed with a calibrated PTW-31010 Semiflex ionization chamber (PTW-Freiburg, Germany). The measured percentage depth dose (PDD) and the dose profiles at the depth of 5cm in a water phantom were compared with the Monte Carlo simulated data. .

In addition, the comparisons were performed with the published clinical data which were based on the Grid block treatments with hole-diameter of 1.0 cm and center-to-center distances of 1.8 cm at the isocenter ^(^[^9^](#_ENREF_9)^,^ [^10^](#_ENREF_10)^)^. Therefore, for these comparisons a similar Grid pattern (i.e. distribution of grid holes) has been used for the simulations. The Grid block was designed to have a hexagonal pattern ^(^[^11^](#_ENREF_11)^)^ with divergent holes. The thickness of the Grid block was chosen to be 7.5 cm of lead. This Grid block was mounted on the block tray holder of the linear accelerator. Figure 2 shows the schematic diagram of the entire pathway of the radiation outside of the water phantom that was used for the simulations in this project.

1. **Therapeutic ratio calculation**

A dose profile from Monte Carlo simulation, across a single hole of the Grid, with 6MV x-ray beam at the depth of 5cm in water phantom has been utilized to calculate therapeutic ratio of the Grid block. In these calculations, it has been assumed that the volume of tissues under each Grid holes could be divided into segments of circular rings shape with 0.1 mm thickness. The tissue cells in these rings are assumed to receive nearly identical irradiation dose (±2%). Equation 1 shows the survival fraction (SF) calculation of the cells under a Grid field using LQ model^(^[^6^](#_ENREF_6)^)^.

 (1)

Where the *Vi* represents the relative number of cells which are receiving a dose ranging from *D_i_* and *D_i+1_*. Figure 3 is a schematic diagram for the beam profile of a single Grid hole.

Assuming the uniform distribution of the cells within the irradiation area, the *Vi* is calculated as the ratio of the area of each ring to the total area under a grid hole.

(2)

*r_max_* is the radius of the largest circle under one hole (Figure 3). This radius is the same as the half of the center-to-center distances between the holes*.*

In Grid therapy, the absorbed dose from a single fraction of open field that creates the same tumor survival fraction as the Grid field is called equivalent uniform dose (EUD) as shown below;

 (3)

Therefore, by taking logarithm (ln) from both side of this equation one can find the Equation 4as: $\alpha_{Tumor} EUD+\beta_{Tumor} EUD^{2}+\ln(SF_{Tumor}(Grid))=0.0$ (4)

The therapeutic advantage of the Grid irradiation was related to increase of the normal tissue survival fractions as a ratio of the normal tissue cell survival fraction under Grid field irradiation to the normal tissue survival fraction under an open field with equivalent dose of EUD, for the same tumor cell survival.

 (5)

$$TR=SF_{Normal}(Grid)/SF_{Normal}(EUD)$$

In the above equation, SF_normal_ (Grid) and SF_normal_ (EUD) are the survival fractions of the normal tissue for the Grid therapy dose and open field with equivalent uniform dose (EUD), respectively.

Recently, some studies have discussed about the suitability of the LQ model when describing cell killing at high doses (>12 Gy). ^(^[^12-14^](#_ENREF_12)^)^. Kirkpatrick^(^[^14^](#_ENREF_14)^)^ has shown that the LQ model underestimates the surviving fraction in the high dose range. However, until now there was no evidence of problems when LQ has been used clinically^(^[^12^](#_ENREF_12)^)^. In this study, in order to validate the radiobiological modeling results, in addition to the LQ model, a Hug–Kellerer (H-K) model ^(^[^15^](#_ENREF_15)^)^ was used to recalculate TR and EUD of the same cell lines for Grid therapy.

The H-K model can be expressed as:

 (6)

Where:

$\alpha=k_{1}-k_{2}.k_{3}$ , $\beta=k_{2}.k_{3}^{2}.(\ln(2)-1/2)/(\ln(2))^{2}$ (7)

In the above equations *k_1_*, *k_2_* and *k_3_* are the parameters of H-K model which can be derived for a tumor type considering its SF2 value, α, and β parameters (Equation 7).

In this project, tumors were divided into 3 groups, based on their radiation sensitivities which is assumed to be directly related to their surviving fraction values for 2Gy dose (SF2). These three groups are composed of; radio-sensitive tumors (SF2<0.4), semi-sensitive tumors (SF2=0.4) and radio-resistant tumors (SF2>0.4). The value of 0.4 has been determined based on the clinical study by Bjork-Eriksson ([16](#_ENREF_16)). In addition, it was assumed that the SF2 value for normal tissue is constant (SF2=0.4). The values of α/β ratios for tumor cells and normal cells were considered to be 10 Gy and 2.5 Gy, respectively^(^[^17^](#_ENREF_17)^)^. The values of the SF2 and α/β ratios for both normal tissue and tumor were used in the linear quadratic model to extract the α and β values (Table 1). The therapeutic ratios are calculated based on the single-fraction Grid therapy. A combination of TR and SF2 values were utilized to evaluate the benefit of the Grid therapy for different tumor histology. In addition, various prescription doses (2 Gy, 10 Gy, 15 Gy and 20 Gy) were used to evaluate relationship between maximum dose and therapeutic ratio.

1. **Clinical reports**

The clinical responses of the patients from different publications, which were treated with Grid therapy, have been used to validate the results of the model introduced in this investigation ^(^[^7^](#_ENREF_7)^,^ [^18^](#_ENREF_18)^,^ [^9^](#_ENREF_9)^,^ [^19^](#_ENREF_19)^,^ [^20^](#_ENREF_20)^,^ [^10^](#_ENREF_10)^)^. Since the correlation of the responses of the Grid therapy with the radiation sensitivity of tumors that were needed from each publication, we had focused on the total responses of the treatments, based on the tumor histology and treatment site. The publications which had compatible results with the objectives of this study are briefly described in Table 2.

In all of the above noted clinical studies, the investigators tried to avoid as much as possible irradiating the normal tissues and critical organs. No margin was added to the GTV and 10Gy to 20Gy were prescribed at the depth of maximum dose using a single treatment field. All of these publications have used similar pattern for the Grid blocks. Their Grid had 1 cm hole diameters with center-to-center spacing of 1.8 cm. However, in the clinical report by Peñagarícano ^(^[^20^](#_ENREF_20)^)^ which was based on MLC system for Grid therapy, the hole spacing was 1cm. All the clinical reports of Grid therapy were refer to curative or palliative advanced tumors (mostly stage III/IV) with sizes larger than 6 cm. The patients in the publications have chemotherapy except in clinical report by Huhn et al ^(^[^7^](#_ENREF_7)^)^ that 7 of 27 patients treated with chemo-radiation in Grid technique for advanced SCC of head and neck (H&N) tumors. Moreover, these patients normally received full standard radiotherapy after a few days from completion of the Grid therapy.

Table 3 shows the list of the SF2 values that were noted in the publications of the clinical data which were mentioned in Table 2.

1. **The Grid design**

The influences of the hole-diameters and center-to-center spacing of the holes in a Grid block on the therapeutic ratio of the Grid therapy were evaluated in these investigations. The dose distributions of a Grid blocks with hole-diameters of 0.5 cm, 0.75 cm, 1.0 cm, 1.25 cm, and 1.5 cm with constant center-to-center spacing of 1.8 cm, were calculated separately using the Monte Carlo simulation technique. Since the prescription dose of 15 Gy per fraction in megavoltage grid therapy is widely accepted ^(^[^9^](#_ENREF_9)^,^ [^19^](#_ENREF_19)^,^ [^21^](#_ENREF_21)^,^ [^10^](#_ENREF_10)^)^, , this dose was considered in comparison of the TR values for different Grid blocks. Equivalent uniform dose (EUD) for a Grid with hole diameter of 1.0 cm and center-to-center distance of 1.8 cm. was calculated. As suggested by Zwicker et al ^(^[^6^](#_ENREF_6)^)^, for all other Grid geometries with different hole diameters and center-to-center distances the maximum doses were adjusted such that the value of the EUD remains fixed.

1. **RESULTS**
2. **Monte Carlo simulation**

Figure 4 shows the comparison between Monte Carlo simulation and measured relative doses along the central axis (PDD) and transverse direction (dose profile) for 6MV x-ray beam. These results show a good agreement between the two sets of data. Simulation has a statistical uncertainty of 1%. More than 90% of the points were passing the gamma comparison to within 3%/3mm clinical criterion.

Figure 5 shows a Monte Carlo simulated 2D dose distribution of a Grid field at the depth of 5 cm in water. The Grid block was design with 1.0 cm hole-diameter and 1.8 center-to-center distances. The valley to peak ratio is about 22%.

1. **Therapeutic ratio calculation**

Figure 6 presents the comparison between the survival fraction of the tumor and normal cells irradiated with Grid technique and open radiation field. These results confirm the therapeutic advantages of Grid therapy compared to conventional radiotherapy regimen. Table 4 shows the therapeutic ratios of the Grid fields, calculated by LQ and H-K models, using the simulated dose profiles as shown on figure 5. These results indicate that the TR values of the radio-resistant tumors are larger than radio-sensitive tumors. In addition, the TR values of the radio-resistant and semi-sensitive tumors, increases by increasing the maximum dose per fraction for Grid therapy. However, no significant changes are seen on the TR values of the radio-sensitive tumors. At the 2Gy dose, there is about 2% difference in therapeutic ratio between tumors with different radio-sensitivities. Therefore, there is no therapeutic advantage of Grid Therapy with 2 Gy maximum doses. In addition, these results demonstrate that the therapeutic advantage of the Grid therapy is more pronounced for radio-resistant tumors. Interestingly, the data in Table 4 indicates that, for 20 Gy dose, the differences between the calculated TR values by the LQ and H-K models for radio-resistant and radio-sensitive tumors, are 1% and 4%, respectively.

1. **Clinical response based on tumor sensitivity**

Figure 7(a) and (b) show the changes of therapeutic ratio as a function of the SF2 values of the tumors, for three different maximum doses of 10 Gy, 15 Gy and 20 Gy, using the LQ and H-K models, respectively. As shown in these figures, the TR value increases by increasing SF2 values (or by decreasing radio-sensitivity). In addition, the differences between the TR values are more apparent at larger doses and in more radio-resistant (higher SF2) tumors. Figure 7(c) presents the relation between the clinical responses of the tumors treated with Grid therapy, using either Grid block or MLC, as a function of SF2 values. This figure shows that the clinical responses of the data are better for tumors with larger SF2 values. These results are consistent with Figure 7a and b. Figure 7(d) indicates the linear function between clinical responses of Grid therapy as a function of model based TR values for 15 Gy Grid therapy and tumor SF2 values. These results are showing how similar these two graphs are (i.e. 1SF2 is equivalent to 3.49 TR).

1. **The Grid design**

Figure 8 shows half of the Monte Carlo simulated dose profiles of Grid-blocks at a depth of 5cm in water phantom. These profiles are calculated for Grids with hole-diameters of 0.5 cm, 0.75 cm, 1.0 cm, 1.25 cm, and 1.5 cm, with a constant center-to-center spacing of 1.8 cm. Similar profiles were created for Grids with different center-to-center spacing.

Figure 9(a) and 9(b) show the impact of the Grid hole-diameter and hole center-to-center spacing on therapeutic ratio of the Grid therapy, respectively. Figure 9(a) indicates that Grid blocks with a hole-diameters of 1.0 cm and 1.25 cm may lead to about 19% higher TR relative to the Grids with hole-diameters smaller than 1.0 cm or larger than 1.25 cm ( with 95% confidence interval).

Figure 9(b) shows that the TR of the Grid block for a given hole diameter remained nearly unchanged (±4%) by increasing the spacing between the Grid holes.

1. **DISCUSSION**

In this study, we have provided the dosimetric simulation of Grid therapy and assessment of the radiobiological responses of tumors with different radio-sensitivities using the LQ and H-K models. The validity of results were examined using the available retrospective clinically reported data. Although the clinical experiences with Grid therapy are limited, an overview of the published reports might provide a guideline for developing a new strategy in this treatment technique. The biological advantages of Grid therapy for different tumor histology have been evaluated. For TRs and EUDs calculations the LQ and H-K models have been used. In addition, the impact of the Grid design as an effective parameter on therapeutic ratio was investigated.

Comparisons between calculated TRs of different tumors and their SF2 values with clinical data indicate the need for a comprehensive guideline/recommendations for tumor selection in order to achieve the optimum treatment result. Although some of the previous clinical and theoretical investigations have demonstrated that it is expected to see a better therapeutic response for radio-resistant tumors in Grid therapy ^(^[^7^](#_ENREF_7)^,^ [^5^](#_ENREF_5)^)^, there was no systematic proof of that. The results of the present study emphasize that even for radio-resistant tumors, clinical responses of Grid therapy may vary with the SF2 value. Some authors ^(^[^22^](#_ENREF_22)^,^ [^23^](#_ENREF_23)^)^ have reported that tumors with different histology may have different SF2 values. Also, they have noted that for a given tumor with a specific histology, there may be different radio-sensitivity characteristics at different anatomical sites. For instance, for Sarcoma tumors, various SF2 values ranging from 0.22 to 0.54 have been introduced. It should be noted that for some tumors such as Melanoma, all cell lines have the SF2 value in radio-resistant range (SF2 > 0.4) ^(^[^24^](#_ENREF_24)^)^. In general, outstanding clinical responses are expected for these types of tumors (i.e. radio-resistant) in Grid therapy. From available clinical data, the local control of 79% in H&N SCC was reported in the Grid therapy technique. In contrast, for conventional fractionation regimen, the most recent and largest randomized trial (RTOG 9003) have reported local-regional control rate of 33-48% for stage III/IV non-metastatic SCC of head and neck cancer ^(^[^25^](#_ENREF_25)^)^. The overall results of this data shown in Figure 7 demonstrate the increase of therapeutic response with SF2 of tumor for both MLC based Grid and block based one.

As shown in Figure 7(a and b), the TR is increasing with prescribed dose in Grid therapy. For a radio-resistant tumor with SF2= 0.55, the calculated TR value for prescribed dose 20 Gy, 15 Gy and 10 Gy was 2.80, 2.03 and 1.45, respectively. However, for a radio-sensitive tumor with SF2=0.28 the calculated TR value for those prescribed doses was 1.15, 1.06 and 0.98, respectively. Therefore, there is not as much benefit of Grid therapy for the radio-sensitive tumors as it is for the radio-resistant tumors.

Comparison between the calculated TRs in Tables 4 and Figure 9 indicates that the radio-sensitivity of tumors and the prescribed dose are more efficient than Grid design in clinical advantages of Grid therapy. From this Monte Carlo study, a Grid designs with the hole diameters between 1.0 cm and 1.25 cm are recommended as they have the optimal TR value. The value of EUD for a Grid with hole diameters of 1.0 cm and a center-to-center distance of 1.8 cm, was 4.41 Gy. In order to compare TRs values for all other Grids with different designs, maximum doses were adjusted in order to maintain EUD constant at 4.41 Gy. In a study ^(^[^6^](#_ENREF_6)^)^ which was about therapeutic advantages of Grid therapy, the authors have worked on one Grid with a specific hole size of 1.3 cm. They calculated EUD for this Grid with hole center-to-center distance of 1.8cm and maximum dose of 15 Gy. They kept EUD value constant to examine TR variations with different hole center-to-center distances. The hole separation distance of 1.7 cm was suggested for their Grid to reach a maximum TR value. The results of our study (Figure 9) also indicate that for a Grid with hole diameter size of 1.25 cm (which is very close to theirs), the center-to-center distance of 1.8 cm has the maximum TR value. We couldn’t find through previous publications a Grid block with hole diameter smaller than 1cm or larger than 1.5 cm to compare the impact of hole diameter and spacing design on their clinical output or therapeutic ratio calculations. Recently, some studies have introduced virtual Grid technique using new technologies.^(^[^26^](#_ENREF_26)^)^ For example recently Jin et al ^(^[^27^](#_ENREF_27)^)^ have introduced a virtual Grid using IMRT technique. They considered a lattice of spheres with diameters of 0.5 cm to create dose distributions of the Grid. But they did not report about their Grid therapeutic ratio calculations or clinical outcome.

One of the challenges that might come is about using a radiobiological model which is more appropriate for high dose per fraction in Grid therapy. Recently a study ^(^[^28^](#_ENREF_28)^)^ has reported a 1% difference in the TR calculation for Grid therapy of Melanoma using both a LQ and MLQ models. This finding is consistent with the results of these investigations. This could be attributed to the small volume of the tissues that receives higher doses under the Grid hole. In addition, the value of the equivalent uniform dose (EUD) in Grid therapy is in the dose range that the LQ model is valid.^(^[^29^](#_ENREF_29)^)^

1. **CONCLUSIONS**

The correlation between the therapeutic ratios of different tumors in Grid therapy with their radiation sensitivities was demonstrated in this project by a LQ and H-K models. These results were then conceded by a comparison with the published clinical data. In this study, it was demonstrated that Grid therapy is most beneficial for the radio-resistant tumors. In addition, there is no clinical benefit of the Grid therapy for the radio-sensitive tumors. Moreover, it has been shown that the Grid blocks with hole diameters ranging from 1.0 to 1.25 may provide the optimum clinical results. The spacing between the Grid holes did not have significant effects on the TR values, but it may impact on selection of the hole diameters (i.e. 1.0 cm spacing between the holes would not permit for the hole diameter of 1.25 cm).

In summary, our results in this study are pointing at the guideline/recommendations as:

1) Knowing the SF2 values of the tumors which are directly related to their radiation sensitivities, could be very helpful in selection of the patients for Grid therapy

2) Grid blocks with hole diameters between 1.0 -1.25 cm could be utilized to achieve an optimum clinical result.

3) Spacing between the holes does not have significant impacts on the biological effect of the Grid.

In our future study, these findings will be tested by the means of in-vitro cell survival experiments using appropriate cell lines.

**ACKNOWLEDGEMENTS**

**REFERENCES**

1. Reddy, S P, R L Hong, S Nagda,B Emami. Effect of tumor bulk on local control and survival of patients with T1 glottic cancer: a 30-year experience. Int J Radiat Oncol Biol Phys. 2007; 69(5): p. 1389-94.

2. Neuner, G, M M Mohiuddin, N Vander Walde, O Goloubeva, J Ha, X Y Cedric,W F Regine. High-dose spatially fractionated GRID radiation therapy (SFGRT): a comparison of treatment outcomes with Cerrobend vs. MLC SFGRT. Int J Radiat Oncol Biol Phys. 2012; 82(5): p. 1642-9.

3. Ha, J K, G Zhang, S A Naqvi, W F Regine,X Y Cedric. Feasibility of delivering grid therapy using a multileaf collimator. Med Phys. 2006; 33(1): p. 76-82.

4. Zhang, H, E L Johnson,R D Zwicker. Dosimetric validation of the MCNPX Monte Carlo simulation for radiobiologic studies of megavoltage grid radiotherapy. Int J Radiat Oncol Biol Phys. 2006; 66(5): p. 1576-83.

5. Zhang, H, J Z Wang, N Mayr, X Kong, J Yuan, N Gupta, S Lo, J Grecula, J Montebello,D Martin. Fractionated grid therapy in treating cervical cancers: Conventional fractionation or hypofractionation? Int J Radiat Oncol Biol Phys. 2008; 70(1): p. 280-8.

6. Zwicker, R D, A Meigooni,M Mohiuddin. Therapeutic advantage of grid irradiation for large single fractions. Int J Radiat Oncol Biol Phys. 2004; 58(4): p. 1309-15.

7. Huhn, J L, W F Regine, J P Valentino, A S Meigooni, M Kudrimoti,M Mohiuddin. Spatially fractionated GRID radiation treatment of advanced neck disease associated with head and neck cancer. Technology in cancer research & treatment. 2006; 5(6): p. 607-12.

8. Collaboration, G S Agostinelli. Geant4-a simulation toolkit. Nuclear Instruments and Methods in Physics Research A. 2003; 506: p. 250-303.

9. Mohiuddin, M, M Fujita, W F Regine, A S Megooni, G S Ibbott,M M Ahmed. High-dose spatially-fractionated radiation (GRID): a new paradigm in the management of advanced cancers. Int J Radiat Oncol Biol Phys. 1999; 45(3): p. 721-7.

10. Sathishkumar, S, S Dey, A S Meigooni, W F Regine, M Kudrimoti, M M Ahmed,M Mohiuddin. The impact of TNF-α induction on therapeutic efficacy following high dose spatially fractionated (GRID) radiation. Technology in cancer research & treatment. 2002; 1(2): p. 141-7.

11. Buckey, C, S Stathakis, K Cashon, A Gutierrez, C Esquivel, C Shi,N Papanikolaou. Evaluation of a commercially-available block for spatially fractionated radiation therapy. Journal of Applied Clinical Medical Physics. 2010; 11(3).

12. Brenner, D J. The linear-quadratic model is an appropriate methodology for determining isoeffective doses at large doses per fraction. in Seminars in radiation oncology. 2008: Elsevier.

13. Chapman, J D C J Gillespie. The power of radiation biophysics—Let's use it. Int J Radiat Oncol Biol Phys. 2012; 84(2): p. 309-11.

14. Kirkpatrick, J P, J J Meyer,L B Marks. The linear-quadratic model is inappropriate to model high dose per fraction effects in radiosurgery. in Seminars in radiation oncology. 2008: Elsevier.

15. Ekstrand, K E. The Hug–Kellerer equation as the universal cell survival curve. PMB. 2010; 55(10): p. N267.

16. Björk-Eriksson, T, C West, E Karlsson,C Mercke. Tumor radiosensitivity (SF 2) is a prognostic factor for local control in head and neck cancers. Int J Radiat Oncol Biol Phys. 2000; 46(1): p. 13-9.

17. Thames, H, S Bentzen, I Turesson, M Overgaard,W Van den Bogaert. Time-dose factors in radiotherapy: a review of the human data. Radiotherapy and Oncology. 1990; 19(3): p. 219-35.

18. Mohiuddin, M, D L Curtis, W T Grizos,L Komarnicky. Palliative treatment of advanced cancer using multiple nonconfluent pencil beam radiation: A pilot study. Cancer. 1990; 66(1): p. 114-8.

19. Mohiuddin, M, J H Stevens, J E Reiff, M S Huq,N Suntharalingam. Spatially fractionated (GRID) radiation for palliative treatment of advanced cancer. Radiation Oncology Investigations. 1996; 4(1): p. 41-7.

20. Peñagarícano, J A, E G Moros, V Ratanatharathorn, Y Yan,P Corry. Evaluation of spatially fractionated radiotherapy (GRID) and definitive chemoradiotherapy with curative intent for locally advanced squamous cell carcinoma of the head and neck: initial response rates and toxicity. Int J Radiat Oncol Biol Phys. 2010; 76(5): p. 1369-75.

21. Reiff, J E, M S Huq, M Mohiuddin,N Suntharalingam. Dosimetric properties of megavoltage grid therapy. Int J Radiat Oncol Biol Phys. 1995; 33(4): p. 937-42.

22. Amundson, S A, K T Do, L C Vinikoor, R A Lee, C A Koch-Paiz, J Ahn, M Reimers, Y Chen, D A Scudiero,J N Weinstein. Integrating global gene expression and radiation survival parameters across the 60 cell lines of the National Cancer Institute Anticancer Drug Screen. Cancer research. 2008; 68(2): p. 415-24.

23. Mundt, A J, A Awan, G S Sibley, M Simon, S J Rubin, B Samuels, W Wong, M Beckett, S Vijayakumar,R R Weichselbaum. Conservative surgery and adjuvant radiation therapy in the management of adult soft tissue sarcoma of the extremities: clinical and radiobiological results. Int J Radiat Oncol Biol Phys. 1995; 32(4): p. 977-85.

24. Marshall, E, J Matthews, J Shaw, J Nixon, P Tumewu, G Finlay, K Holdaway,B Baguley. Radiosensitivity of new and established human melanoma cell lines: comparison of [3 H] thymidine incorporation and soft agar clonogenic assays. European Journal of Cancer. 1994; 30(9): p. 1370-6.

25. Fu, K K, T F Pajak, A Trotti, C U Jones, S A Spencer, T L Phillips, A S Garden, J A Ridge, J S Cooper,K K Ang. A Radiation Therapy Oncology Group (RTOG) phase III randomized study to compare hyperfractionation and two variants of accelerated fractionation to standard fractionation radiotherapy for head and neck squamous cell carcinomas: first report of RTOG 9003. Int J Radiat Oncol Biol Phys. 2000; 48(1): p. 7-16.

26. Zhang, X, J Penagaricano, Y Yan, S Sharma, R Griffin, M Hardee, E Han,V Ratanatharathom. Application of Spatially Fractionated Radiation (GRID) to Helical Tomotherapy using a Novel TOMOGRID Template. Technology in cancer research & treatment. 2014: p. tcrtexpress. 2013.600261.

27. Jin, J-Y, B Zhao, J M Kaminski, N Wen, Y Huang, J Vender,I J Chetty. A MLC-based inversely optimized 3D spatially fractionated grid radiotherapy technique. Radiotherapy and Oncology. 2015.

28. Zhang, H, H Zhong, R F Barth, M Cao,I J Das. Impact of dose size in single fraction spatially fractionated (grid) radiotherapy for melanoma. Med Phys. 2014; 41(2): p. 021727.

29. Gholami, S, H Nedaie, A Meigooni,F Longo. Grid therapy: impact of radiobiological models on calculation of therapeutic ratio. in World Congress on Medical Physics and Biomedical Engineering, June 7-12, 2015, Toronto, Canada. 2015: Springer.

30. Björk-Eriksson, T, C M West, E Cvetskovska, M Svensson, E Karlsson, B Magnusson, N J Slevin, S Edström,C Mercke. The lack of correlation between proliferation (Ki-67, PCNA, LI, Tpot), p53 expression and radiosensitivity for head and neck cancers. British journal of cancer. 1999; 80(9): p. 1400.

31. Weichselbaum, R R, M A Beckett, J L Schwartz,A Dritschilo. Radioresistant tumor cells are present in head and neck carcinomas that recur after radiotherapy. Int J Radiat Oncol Biol Phys. 1988; 15(3): p. 575-9.

32. Weichselbaum, R R, M A Beckett, M A Simon, C McCauley, D Haraf, A Awan, B Samuels, J Nachman,A Dritschilo. In vitro radiobiological parameters of human sarcoma cell lines. Int J Radiat Oncol Biol Phys. 1988; 15(4): p. 937-42.

33. Haikonen, J, V Rantanen, K Pekkola, J Kulmala,R Grénman. Does skin fibroblast radiosensitivity predict squamous cancer cell radiosensitivity of the same individual? International journal of cancer. 2003; 103(6): p. 784-8.

34. Guerrero, M X A Li. Extending the linear–quadratic model for large fraction doses pertinent to stereotactic radiotherapy. PMB. 2004; 49(20): p. 4825.

35. Deschavanne, P J B Fertil. A review of human cell radiosensitivity in vitro. Int J Radiat Oncol Biol Phys. 1996; 34(1): p. 251-66.

36. Rödel, C, J Haas, A Groth, G G Grabenbauer, R Sauer,F Rödel. Spontaneous and radiation-induced apoptosis in colorectal carcinoma cells with different intrinsic radiosensitivities: survivin as a radioresistance factor. Int J Radiat Oncol Biol Phys. 2003; 55(5): p. 1341-7.

**FIGURES**


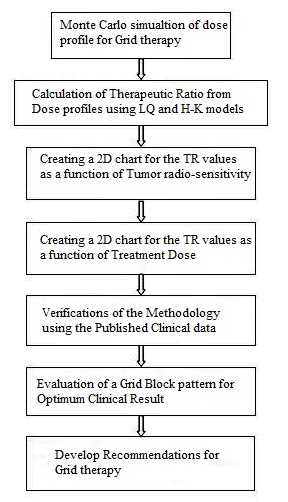


FIG. 1. The flow chart of the process of this project.


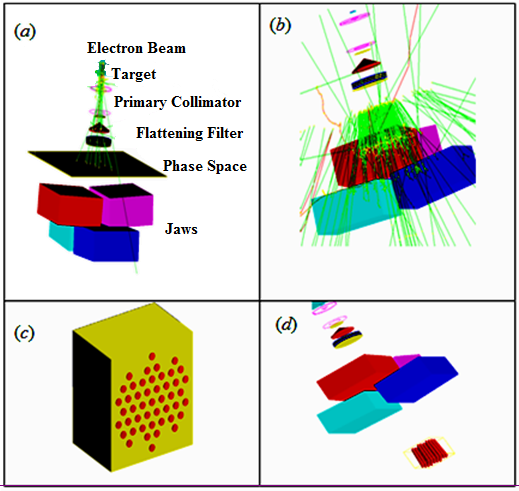


FIG. 2. Step one in medical linac simulation using phase space (Phs), different parts in the simulation are represented: electron beam, target, primary collimator, beryllium window, flattening filter, Ion chamber, phs and Jaws (a). Step two in simulation using Phs as a source (b), the hexagonal pattern of modeled Grid block (c), The schematic diagram of the modeled linear accelerator with a Grid (d).


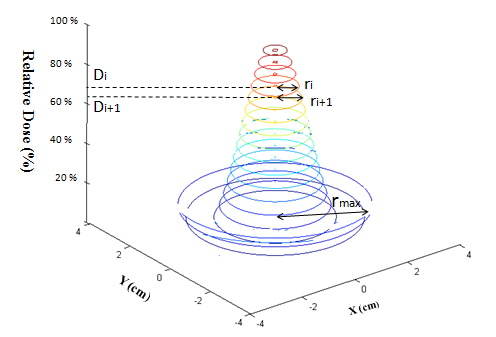


FIG. 3. Schematic diagram of the dose profile under a single hole in a GRID field with the related parameters that were considered for TR calculations.


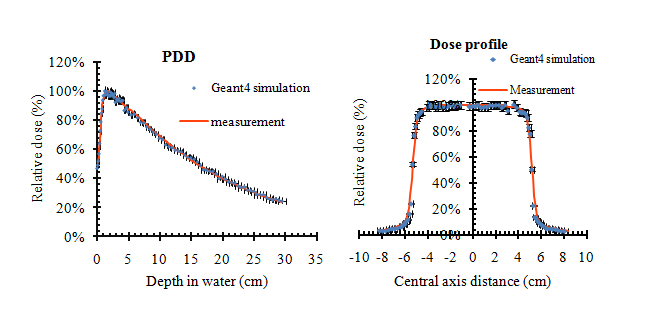


FIG. 4. A comparison between the simulated and measured PDD and dose profile of 6MV x-ray beam (with 3% error bar).


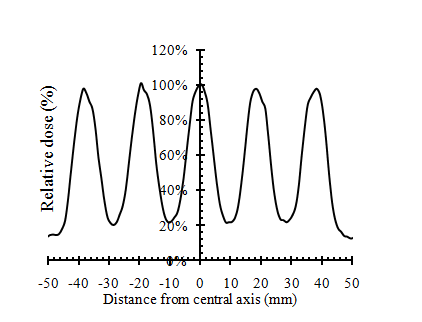


FIG. 5. The Monte Carlo simulated beam proﬁle of a 6-MV spatially fractionated photon beam at 5 cm depth in water.


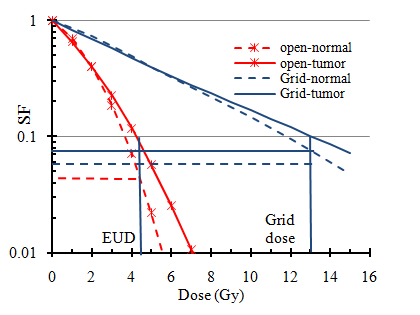


FIG. 6. The horizontal solid blue line shows SF value for the tumor. The cross point of this line with the survival curve of the tumor with open field reflect the equivalent uniform dose. The horizontal blue dashed line indicates SF for the normal cell in Grid technique. The horizontal red dashed line indicates SF for the normal cell in an open field. The ratio of the survival fraction of normal cells with Grid therapy to the open field is called therapeutic ratio.


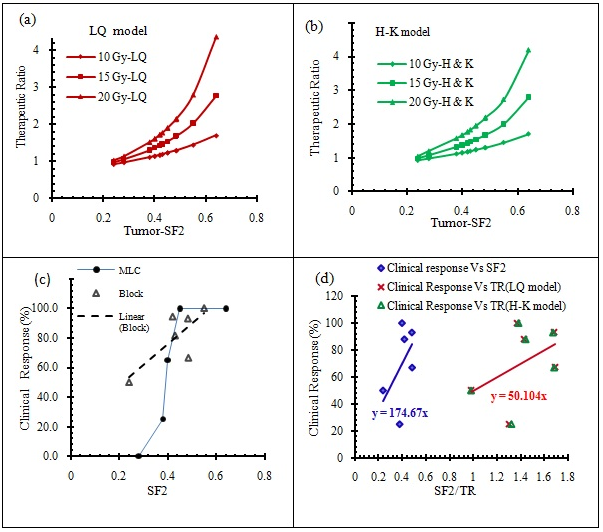


FIG. 7. comparison between therapeutic ratios for different maximum doses as a function of the tumor survival fraction at 2 Gy dose (SF2),using LQ model (a) and H-K model (b), clinical response of different tumors as a function of SF2 for blocked Grid (open symbol) and MLC (solid Symbol) (c). Clinical response of Grid therapy from publications versus calculated TRs and tumors SF2 value (d).


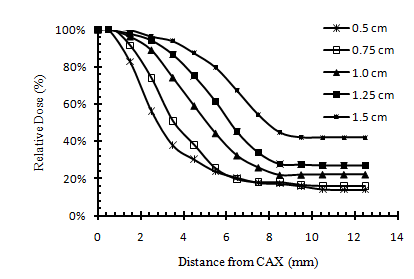


FIG. 8. Comparison of the Monte Carlo simulated dose semi-profiles of the Grid blocks with different hole-diameters, at a depth of 5cm in water.


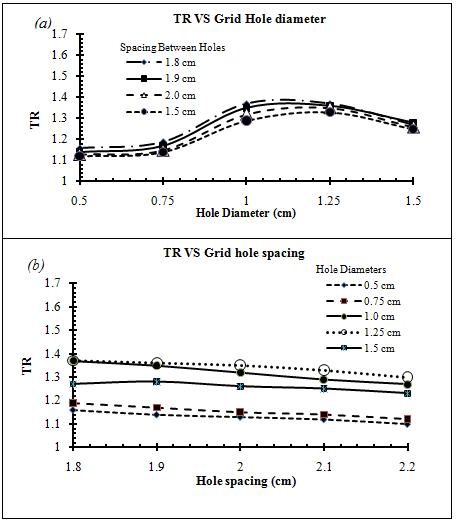


FIG. 9. Influence of Grid hole-diameter (a) and center-to-center spacing, on the therapeutic ratio (TR) (b).

**Figure legends**

FIG. 1. The flow chart of the process of this project.

FIG. 2. Step one in medical linac simulation using phase space (Phs), different parts in the simulation are represented: electron beam, target, primary collimator, beryllium window, flattening filter, Ion chamber, phs and Jaws (a). Step two in simulation using Phs as a source (b), the hexagonal pattern of modeled Grid block (c) , The schematic diagram of the modeled linear accelerator with a Grid (d).

FIG. 3. Schematic diagram of the dose profile under a single hole in a GRID field with the related parameters that were considered for TR calculations.

FIG. 4. A comparison between the simulated and measured PDD and dose profile of 6MV x-ray beam (with 3% error bar).

FIG. 5. The Monte Carlo simulated beam proﬁle of a 6-MV spatially fractionated photon beam at 5 cm depth in water.

FIG. 6. The horizontal solid blue line shows SF value for the tumor. The cross point of this line with the survival curve of the tumor with open field reflect the equivalent uniform dose. The horizontal blue dashed line indicates SF for the normal cell in Grid technique. The horizontal red dashed line indicates SF for the normal cell in an open field. The ratio of the survival fraction of normal cells with Grid therapy to the open field is called therapeutic ratio.

FIG. 7. comparison between therapeutic ratios for different maximum doses as a function of the tumor survival fraction at 2 Gy dose (SF2),using LQ model (a) and H-K model (b), clinical response of different tumors as a function of SF2 for blocked Grid (open symbol) and MLC (solid Symbol) (c). Clinical response of Grid therapy from publications versus calculated TRs and tumors SF2 value (d).

FIG. 8. Comparison of the Monte Carlo simulated dose semi-profiles of the Grid blocks with different hole-diameters, at a depth of 5cm in water.

FIG. 9. Influence of Grid hole-diameter (a) and center-to-center spacing, on the therapeutic ratio (TR) (b).

**TABLES**

TABLE 1. α and β values of tumor and normal cells for three different types of tumors cells.

|  | *SF_2_(Tumor)*  *[α/β (Tumor)=10]* | *α_(Tumor)_* | *β_(Tumor)_* | *SF_2_(Normal)*  *[α/β (normal)=2.5]* | *α_(normal)_* | *β_(normal)_* |
| --- | --- | --- | --- | --- | --- | --- |
| *Radio-Sensitive Tumor* | 0.2 | 0.670 | 0.067 | 0.4 | 0.254 | 0.101 |
| *Semi-Sensitive Tumor* | 0.4 | 0.381 | 0.038 | 0.4 | 0.254 | 0.101 |
| *Radio-Resistant Tumor* | 0.5 | 0.288 | 0.028 | 0.4 | 0.254 | 0.101 |

TABLE 2. Clinical total response of Grid therapy for tumors with different histological characteristics and treatment sites. The numbers in the parantesis for each tumor type represent the total response under Grid therapy.

| *References* | *Tumor histology or site* | | | | |
| --- | --- | --- | --- | --- | --- |
| *Mohiuddin et al*.^(^[^18^](#_ENREF_18)^)^ | Osteosarcoma  (100%) | Liposarcoma  (50%) | Leiomyosarca  (100%) | Colorectal  (100%) |  |
| *Mohiuddin et al*.^(^[^19^](#_ENREF_19)^)^ | Sarcoma  (94%) | SCC  (92%) | Melanoma  (83%) | Adenocarcinoma  (69%) |  |
| *Mohiuddin et al*.^(^[^9^](#_ENREF_9)^)^ | Sarcoma  (83%) | SCC  (94%) | Adenocarcinoma  (94%) | Melanoma  (50%) |  |
| *Sathishkumar et al*.^(^[^10^](#_ENREF_10)^)^ | SCC  (100%) | Adenocarcinoma  (More that 90%) | Melanoma  (More than 80%) |  |  |
| *Huhn et al*.^(^[^7^](#_ENREF_7)^)^ | SCC of H &N  (93%) | |  |  |  |
| *Peñagarícano et al*.^(^[^20^](#_ENREF_20)^)^ | Parotid  (0%) | Base of tongue  (30%) | Maxillary sinus  (50%) | Nasopharynx,  Retromolar trigone,  and Larynx  (100%) | Tonsil  (25%) |

TABLE 3. SF2 value for different tumors and their corresponding references.

| *Tumor histology* | *Larynx* | *Base of tongue* | *Nasopharynx* | *Retromolar trigone* | *Parotid* | *Sarcoma* | *Melanoma* |
| --- | --- | --- | --- | --- | --- | --- | --- |
| *SF2*  *Reference* | 0.45 | 0.40 | 0.45 | 0.64 | 0.28 | 0.42 | 0.48 |
|  | ([30](#_ENREF_30)) | ([31](#_ENREF_31)) | ([30](#_ENREF_30)) | ([31](#_ENREF_31), [32](#_ENREF_32)) | ([33](#_ENREF_33)) | ([23](#_ENREF_23)) | ([34](#_ENREF_34)) |
| *Tumor histology* | *SCC* | *Adenocarcinoma* | *Osteosarcoma* | *Liposarcoma* | *Leiomysarcoma* | *Colorectal* | *Tonsil* |
|  |  |  |  |  |  |  |  |
| *SF2* | 0.48 | 0.40 | 0.42 | 0.24 | 0.55 | 0.40 | 0.38 |
| *Reference* | ([34](#_ENREF_34)) | ([34](#_ENREF_34)) | ([35](#_ENREF_35)) | ([23](#_ENREF_23)) | ([32](#_ENREF_32)) | ([36](#_ENREF_36)) | ([31](#_ENREF_31)) |

TABLE 4. Therapeutic ratio of different tumor types as a function of the maximum dose.

| *Tumor type* | *LQ model* | | | | *H-K model* | | | |
| --- | --- | --- | --- | --- | --- | --- | --- | --- |
|  | *2 Gy* | *10 Gy* | *15 Gy* | *20 Gy* | *2 Gy* | *10 Gy* | *15 Gy* | *20 Gy* |
| *Radio-sensitive* | 0.97 | 0.88 | 0.90 | 0.94 | 0.97 | 0.88 | 0.91 | 0.98 |
| *Semi sensitive* | 0.99 | 1.15 | 1.37 | 1.62 | 0.99 | 1.15 | 1.38 | 1.66 |
| *Radio-resistant* | 0.99 | 1.34 | 1.76 | 2.28 | 0.99 | 1.34 | 1.75 | 2.3 |

**Table captions**

TABLE 1. α and β values of tumor and normal cells for three different types of tumors cells.

TABLE 2. Clinical total response of Grid therapy for tumors with different histological characteristics and treatment sites.

TABLE 3. SF2 value for different tumors and their corresponding references.

TABLE 4. Therapeutic ratio of different tumor types as a function of the maximum dose.
